# High performance computing environment for multidimensional image analysis

**DOI:** 10.1186/1471-2121-8-S1-S9

**Published:** 2007-07-10

**Authors:** A Ravishankar Rao, Guillermo A Cecchi, Marcelo Magnasco

**Affiliations:** 1 IBM T.J. Watson Research Center, Yorktown Heights, NY 10598, USA; 2 Rockefeller University, 1230 York Avenue, New York, NY 10021, USA

## Abstract

**Background:**

The processing of images acquired through microscopy is a challenging task due to the large size of datasets (several gigabytes) and the fast turnaround time required. If the throughput of the image processing stage is significantly increased, it can have a major impact in microscopy applications.

**Results:**

We present a high performance computing (HPC) solution to this problem. This involves decomposing the spatial 3D image into segments that are assigned to unique processors, and matched to the 3D torus architecture of the IBM Blue Gene/L machine. Communication between segments is restricted to the nearest neighbors. When running on a 2 Ghz Intel CPU, the task of 3D median filtering on a typical 256 megabyte dataset takes two and a half hours, whereas by using 1024 nodes of Blue Gene, this task can be performed in 18.8 seconds, a 478× speedup.

**Conclusion:**

Our parallel solution dramatically improves the performance of image processing, feature extraction and 3D reconstruction tasks. This increased throughput permits biologists to conduct unprecedented large scale experiments with massive datasets.

## Background

Progress in biology is dependent on the ability to observe, measure and model the behavior of organisms at multiple levels of abstraction, from the microscopic to the macroscopic. There has been a tremendous growth recently in the techniques to probe the structure and workings of cellular and organ-level mechanisms. Significant advances have been made in areas such as serial block face microscopy [1], and knife-edge microscopy [2] that allow microstructure information to be gathered at unprecedented levels of both detail and scope. At the same time, advances have also been made in gathering temporal image data streams from microscopic samples with the use of fluorescent and multi-photon imaging techniques [3]. The increasing spatial and temporal resolution available, combined with advanced sectioning techniques are providing extremely content-rich data to biologists and puts unprecedented power in their hands.

This is a game-changing development, since biologists are no longer limited to carrying out experiments to test a single hypothesis at a time. They are now able to vary multiple parameters simultaneously, and observe several phenomena of relevance using multi-spectral techniques. This can be combined with recent advances in data mining techniques to determine relationships and correlations amongst the many variables of interest. This allows a significantly larger parameter space to be explored.

However, there is a mounting problem being faced by practitioners and researchers today, which is the computational bottleneck: the data storage and processing needs are growing exponentially. It may take several hours or even days to process the collected data, and the resulting throughput time may be unacceptable to support desired workflows in laboratories. Unless the computational issues are addressed immediately, biologists will be overwhelmed with the data collected, and will not have adequate tools to process and extract meaning from the data. Though computer vision techniques have been applied in the past to partially automate some of the analysis (e.g. [3]), the current challenge is to process much larger quantities of data (several gigabytes typically) with sufficiently high throughput. This would allow biologists to interpret experimental results rapidly and ideally in an interactive fashion.

A related problem is that of building models from the collected data, which is a useful technique to test the understanding of the phenomena of interest. As the data expose interactions at finer spatial and time scales, the variables that are modeled also increase in number and complexity. This increases the computational burden on the modeling effort as well.

We present a solution to this challenge, which is based on a high-performance computing (HPC) architecture. An example of this is IBM's Blue Gene Supercomputer. There has been a long history of using HPC to model problems in physics, but its use in biology has been very recent and rather limited. In general, HPC has not been used much in bio-imaging applications due to the difficulty in porting code to parallel machines. Algorithms for image processing, such as segmentation and feature extraction are not being sufficiently developed and investigated in a HPC context. Though there was interest in this area in the mid-1990s [4], this appears to have waned, and the use of HPC for imaging applications is currently quite limited.

However, the landscape is rapidly changing due to the increased availability of HPC platforms, improvements in parallel programming environments (such as the emergence of the Message Passing Interface as a standard), and the availability of toolkits to perform parallel data mining. HPC has significant potential to be applied to problems in biology, and in microscopy imaging applications in particular. The high computational demands of simulation and modeling complex systems can also be addressed through HPC. So a single HPC architecture can support multiple computational requirements, ranging from analyzing data to building and simulating models.

### Requirements

We now examine the computational requirements for a total system dedicated to handle biological imaging tasks. The following tasks would need to be performed.

(1) **Data collection**: the system needs to gather and store the images acquired (2) **Deconvolution**: the acquired images may contain distortions such as blur that occur in the front end optics (3) **Segmentation and feature extraction**: the raw images need to be segmented into regions of interest and processed to extract domain-specific features that aid in classification (4) **Analysis and interpretation**: the segmented regions are interpreted as parts of a larger biological structure such as a neuron or organ. (5) **Modeling and prediction**: models for function (e.g. neural connectivity models) can be built to predict the behavior of entities of interest. This phase may require the use of simulation and optimization techniques. From the list of tasks above, the following core computational requirements can be identified (1) The ability to handle large data sets, including storage and retrieval. (2) High throughput processing is required. (3) The visualization of results is important.

As a case study, we consider the system developed by Denk and Horstmann [1], that consists of a serial block-face scanning electron microscope (SEM) to explore 3-d connectivity in neural tissue. Light microscopy is incapable of resolving the fine structure such as dendrites, which necessitates the use of a SEM. The system is able to obtain slices that are 50 nm thickness, with a 50 × 40 *μ*m area, and 27 nm pixel size. This results in single images of size 4 megabytes. Typically, 2000 slices are obtained, giving rise to a stack of 8 GB of data. Several such stacks need to be collected to gain information about neural connectivity in a functional area of the brain. The connectivity within the neural tissue is inferred by identifying structures within the 2D slices, such as neurites, and tracking them across successive slices. This permits the reconstruction of a 3D model of structures of interest, such as a neuron with its soma, dendrites and axon. The ultimate use of such reconstructed structures would be in developing accurate computational models of cortical function.

The system developed by Denk and Horstmann [1] operates at the nanometer scale. A similar system developed by McCormick *et al *[2] operates at the micrometer scale, and can section an entire mouse brain. The computational requirements arising from both systems are very similar. The need for time-varying image analysis arises from biological experiments such as the analysis of macrophage images from the BioImaging Institute at MIT [3]. The goal here is to observe the cell motility of macrophages under different ambient conditions. This requires 3D deconvolution to be performed on a sequence of images. Again, the computational needs of such experiments are enormous.

### High-throughput processing

In this paper, we restrict our scope to solving the problem of high-throughput processing. Since the performance of single CPU machines is not sufficient to handle the size of the case-study data set described above, the use of parallel processing is inevitable. We examine the characteristics of our case-study data set.

1. Significant communication is required between processors. This is specifically true of image processing tasks.

2. The bulk of the communication is between nearest neighbors.

3. Computation and communication needs are well balanced. For instance, consider the operation of recursive 3D median filtering, which is useful for combating noise. Every iteration of the filtering operation may require significant amount of data communication. Suppose we use 1024 processors for the 8 GB stack, with each processor storing 8 MB of data. Assuming up to half this data needs to be communicated, we have a communication need of 4 MB per process to be sent/received to/from its 26 neighbors. This is a large amount of data especially since it may need to be communicated at every iteration of the computation.

There are many possible ways of using parallel processing systems to meet these requirements, such as clusters of workstations, shared memory systems and the use of game processors. We chose to implement our system on the IBM Blue Gene supercomputer due to its scalability (to upwards of 100,000 processors) and its specialized interconnect technology, offering superior communication speeds.

## Results

In order to make advances in microscopy, progress needs to be made on several fronts simultaneously, including new methods for image acquisition, processing algorithms for feature extraction and analysis, and the computational architecture and methodology for fast processing. The focus of this paper is on the latter issue, and deals with the use of parallel computation to address the throughput requirements. There are other research efforts to investigate algorithms for feature extraction and reconstruction [5]. Our goal in this paper has been to show how such algorithms can be implemented on a parallel architecture.

**Figure 1:**
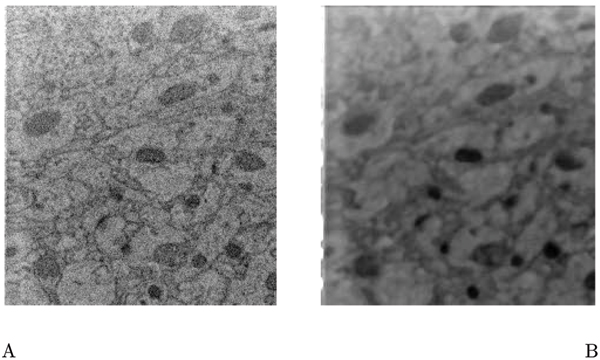
The result of applying 3D median filtering using a kernel size of 5 × 5 × 5 pixels. (A) Shows the original data, of size 234 × 256 pixels, where each pixel is 27 nm. (B) Shows the median filtered data. The filtering eliminates the fine structure in the image, which can be considered noise for the purpose of contour extraction. However the major contours are well preserved

**Figure 2:**
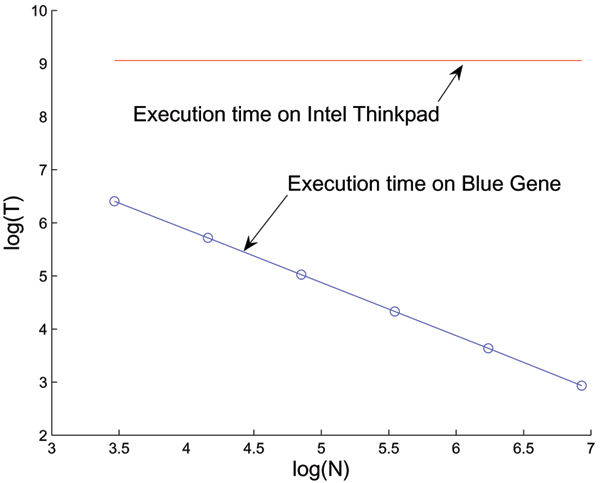
Comparison of execution times. The blue line at the bottom indicates the computation time for 3D median filtering as a function of the number of processors used. As the number of processors increased from 32 to 1024, the computation time decreased from 606 to 18.8 seconds. A logarithmic scale is used for both axes. The red line on the top shows the computation time for the same task running on a 2 GHz Intel processor inside an IBM Thinkpad machine. Clearly, several orders of magnitude speedup can be achieved in a multiprocessor environment.

Our system is written using MPI (message passing interface). We present results of using our system to process images from the serial block-face SEM apparatus [1] on an IBM Blue Gene supercomputer. As an illustration, we implemented recursive 3D median filtering on Blue Gene, and found that a single pass of a 5 × 5 × 5 filtering operation takes 18.8 seconds for 64 slices of 2048 × 1872 grayscale images on 1024 processors arranged in an 8 × 8 × 16 Cartesian grid. The result is shown in Figure 1. The same computation running in MATLAB on a single Intel 2 GHz processor took 143 minutes, or about 456 times slower. This illustrates that the use of HPC can have a dramatic improvement in the throughput of image processing tasks, and that HPC has tremendous potential to influence the fields of bio-imaging and microscopy. Figure 2 depicts the speedup achieved as the number of processors is increased from 32 to 1024 for the 3D median filtering task. Clearly, one can obtain real-time performance for this task on a multiprocessor machine with sufficient number of processors. This would be extremely beneficial for interactive data analysis, algorithm development and visualization of the entire data set.

**Figure 3:**
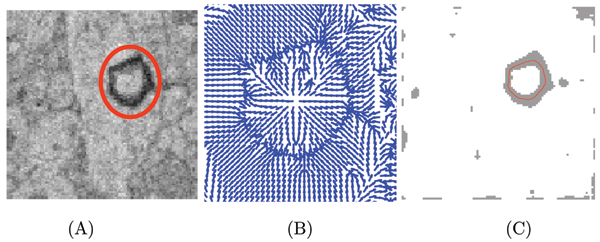
Finding contours of objects (neural structures). (A) The original image. The contour that needs to be extracted is encircled in red, which forms the initial snake. The image size is 101 × 101 pixels, with each pixel of size 27 nm. (B) An expanded image showing the normalized gradient vector field around the contour. Note that the vectors indicate the direction of deformation of the snake as it accommodates itself to the shape of the desired contour. (C) The final result, showing the snake acquires the contour shape of the neural structure.

We implemented the technique described by Xu and Prince [6] for contour processing. An initial contour is selected by the user, which encircles the object of interest, say a neural structure, such as a portion of a dendrite as shown in Figure 3. The snake, a deformable template, then accommodates itself to fit the exact contour of the dendritic structure. This reduces the burden on the user in specifying the detailed contour.

**Figure 4:**
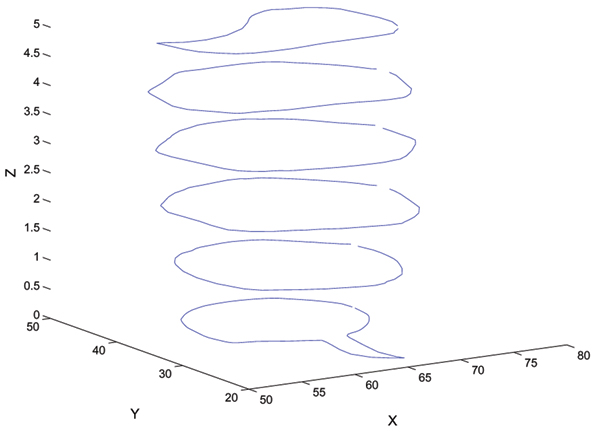
Finding contours of objects. This plot shows the contour of a neural structure obtained from successive slices. The contour in the first slice was initialized by manually drawing a circle around the neural structure, and then refined with the snake algorithm. The subsequent contours are initialized with the final contour of the previous slice, and then deformed to conform to the image data. The X and Y axis units are in pixels, and the Z axis represents the index number of each slice.

This procedure is performed on the first image slice. The next image slice uses the snake from the previous slice to initialize the contour, and executes the same energy-minimizing algorithm as before. This procedure is repeated across multiple image slices. The 2D contours extracted can be assembled into a 3D model of neural connectivity. A partial reconstructed model is shown in Figure 4. This shows a part of a dendrite that has been detected and rendered using the technique described above. There are possibly hundreds of such structures within a given slice of neural tissue. We have chosen to illustrate the reconstruction of a small portion of the data, as our work is at an early stage, and further results will be forthcoming.

**Figure 5:**
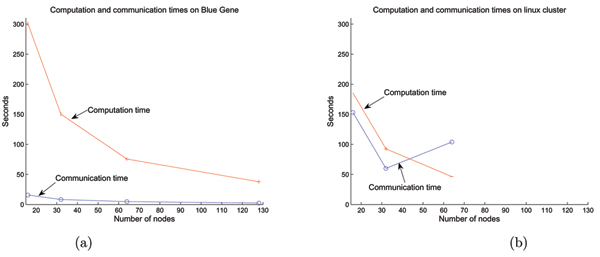
Comparison of algorithm performance on different platforms. (a) On the Blue Gene/L platform, the communication time is a small fraction of the computation time. (b) On the Intel Linux cluster, the communication time is of the same order of the computation time.

In Figure 5 we compare the performance of our algorithm running on Blue Gene/L versus an Intel Linux cluster. No changes to the MPI program were required, and it was merely recompiled on the Linux cluster. The cluster used upto 64 nodes of Intel Pentium III CPUs running at 1.3 GHz and connected via 100 Mb/s ethernet. The communication time in Blue Gene for the 3D median filtering task is approximately 5% of the total time. Whereas, for the Linux cluster, the communication time is more than 40% of the total time. The problem size remained fixed.

**Figure 6:**
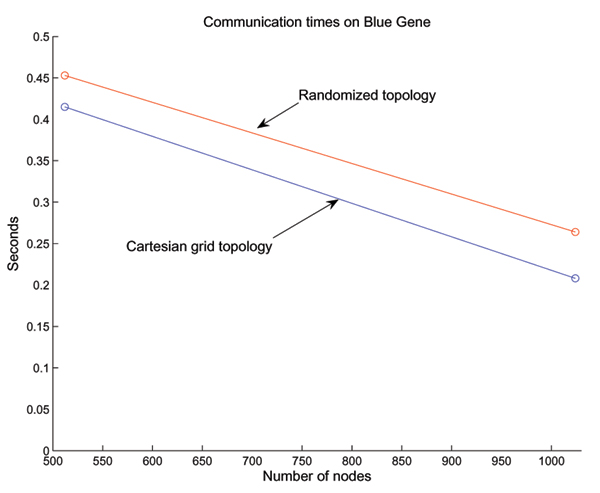
Comparison of communication times on Blue Gene/L. The communication using the cartesian grid mapping is more efficient than using a randomized mapping.

The 3D torus interconnection architecture on Blue Gene/L is used in conjunction with other schemes such as tree-based networks to optimize communication times. In order to demonstrate the efficiency of the torus network with dedicated nearest-neighbor connectivity, we performed the following experiment. We ran the 3D median filtering algorithm in two modes. The first mode used a cartesian mapping to the torus, such that the ranks were sequentially assigned in a 3D grid in correspondence with the 3D torus on the machine. The second mode used a randomized mapping, where this orderly correspondence between ranks and the torus did not exist. Figure 6 shows that the randomized mapping was 10% slower for a 512 node configuration, and 30% slower for a 1024 node configuration.

## Discussion

We expect that other image processing tasks, such as iterative morphological operations (e.g. dilations followed by erosions), or recursive filtering (e.g. recursive 3D median filtering) will also benefit from implementation on an HPC platform if they operate on large datasets. In general, any local neighborhood operation can be computed advantageously using our method as shown in Figure 2.

The procedure described to produce the result in Figure 4 is semi-automated. There are other approaches in the literature which are more sophisticated, such as the technique developed by Busse *et al *[5], which is also semi-automatic. We have chosen to explore a parallel processing solution to the reconstruction problem, and are initially implementing simpler approaches.

Figure 5 shows that the Blue Gene/L communication network is superior to that of the Linux cluster used, and that the communication overhead scales with the number of processors. The Linux cluster did not exhibit appropriate scaling behavior due to the configuration of the switches, which were likely deployed in 32 node banks.

Other studies by Almasi *et al. *have demonstrated that a wide variety of scientific applications can scale up to tens of thousands of processors on Blue Gene/L [7]. We view this as a significant advantage in using the Blue Gene/L platform. The image processing application can be written once using MPI, and the hardware platform provides the desired scaling.

The significance of the result in Figure 6 depends on the precise task being performed. If the task is computation bound, then improvements in communication may not have a significant effect on the throughput. However, for communication-bound tasks, the demonstrated improvement of the cartesian mapping may be significant.

Other studies, such as Agarwal *et al*. [8] have also demonstrated an increase in communication times when the task is not optimally mapped to the processors on Blue Gene/L. This shows that the mapping of communicating objects to nearby processors is desirable.

To summarize this discussion, it is advantageous to use an HPC architecture that optimizes nearest-neighbor communication on a 3D grid. Depending on the task to be performed, this communication efficiency may result in significant throughput increases as compared with other network topologies. The comparisons shown in this paper are not meant to be comprehensive, and the benchmarking of image processing applications on HPC platforms needs further investigation. This may require a community-wide effort to create and measure appropriate benchmarks on multiple HPC platforms. As an example, NIST has been conducting a series of TRECVID workshops on measuring video image database retrieval performance for several years [9].

Our system has been designed with MPI, a *de-facto *standard environment for parallel processing. This enables the system to be used widely across different platforms. Given the increasing popularity of grid computing, and the increasing availability of supercomputers, we expect to see wider usage of parallel processing techniques in areas such as microscopy. In order to fully exploit the available parallel platforms, we recommend that students at universities should be trained to use such technology. Furthermore, curricula in courses such as image processing and computer vision should cover parallel processing techniques.

## Conclusion

Our results demonstrate that the use of HPC can have a dramatic improvement in the throughput of image processing tasks, and that HPC has tremendous potential to influence the fields of bio-imaging and microscopy.

The main contribution of this paper is to develop a parallel image processing system for handling multidimensional image data that optimizes computation and communication needs in a multiprocessor system. This is achieved through an appropriate domain decomposition that exploits support in MPI for computation in cartesian grids.

Our results show that by using the Blue Gene/L machine, significant throughput increases can be achieved compared to conventional clusters. Furthermore, the domain decomposition and algorithms presented in this paper show favorable scaling behavior as the number of processors is increased.

This paper presents early implementation results, and further work needs to be done to incorporate more sophisticated image processing algorithms in this environment. Additionally, time-domain processing capability needs to be added.

## Methods

**Figure 7:**
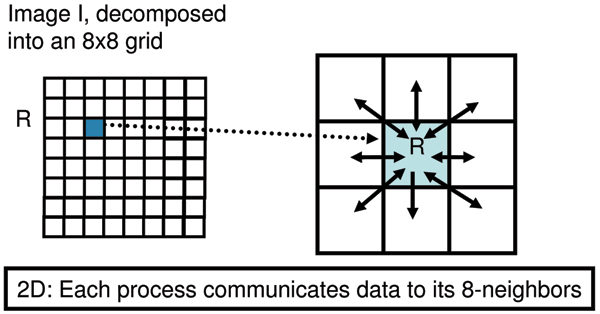
Domain decomposition for a 2D image. The image is divided into 8 × 8 tiles. The tile **R **communicates data to its 8 neighbors.

**Figure 8:**
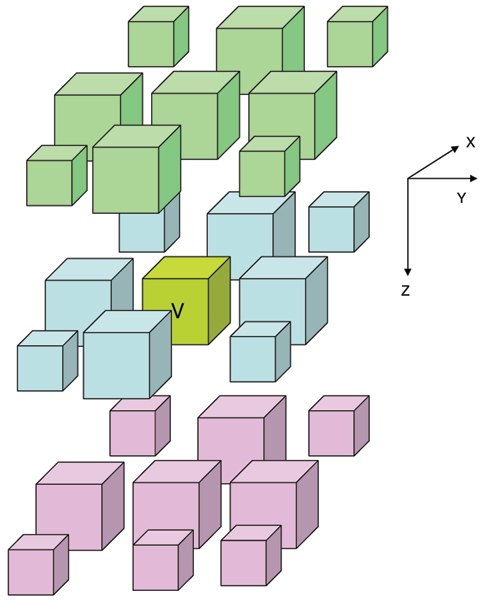
Domain decomposition for a 3D image using a 3D Cartesian grid. Each unit communicates with 26 neighbors. Both send and receive commands are required. This communication cost may need to be incurred at every iteration of an algorithm (e.g. recursive median filtering).

We propose an image processing system architecture specially designed to leverage an HPC platform. A challenge in parallelizing image processing tasks is to arrive at the proper domain decomposition, which specifies how the task is to distributed among the various processors available. We perform an appropriate domain decomposition by assigning processors to spatial 3D volume elements. The original spatial 3D image is broken down into 3D volume elements, where each volume element is assigned to a processor. Each processor communicates information with its nearest neighbors, as shown in Figures 7 and 8. This reduces communication overhead, while balancing the load across the different processors.

### Proposed Solution

Based on the requirements in the background section, we propose the following solution. We use MPI (Message Passing Interface), a widely used message passing library standard. There are several open source implementations available for different computing platforms.

**Figure 9:**
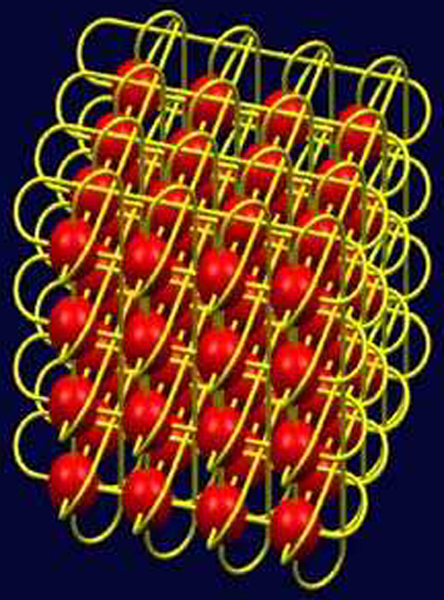
The 3D torus architecture of the IBM Blue Gene/L machine. There are dedicated interconnections between nearest neighbors on a 3D toroidal grid. This minimizes communication overhead between nearest neighbors, and is an ideal solution for 3D image processing tasks.

Due to the 3D geometry of the problem, we use a Cartesian grid for domain decomposition, which is supported by an MPI mechanism for defining local process topologies through the MPI_CART_CREATE command. The MPI Cartesian grid communicator is optimum for nearest-neighbor communications, is architecture independent and works best with hardware-level support. This communicator is described by the *MPI_CART_GRID *command, and serves as a hint to the MPI implementation that sequential process numbers should correspond to locations on a 3D grid. (MPI assigns numbers to each process, and in general there is no restriction on which physical processor runs a given process. This may be acceptable if the processors are physically located on distributed computers such as in a grid. However, if the hardware permits, it is advantageous to cluster the processes tightly as in a cartesian grid). This support is provided in the IBM BlueGene machine/L [10] through the use of a 3D torus architecture, as described in Figure 9. This architecture optimizes nearest-neighbor communication.

The compute nodes in Blue Gene/L are interconnected through multiple complementary high-speed low-latency networks, including a 3D torus network. We directly map the total 3D image volume that needs to be processed onto a 3D torus. This is done by partitioning the 3D image volume into the number of processors available, and ensuring that neighboring processors on the Blue Gene/L machine are dedicated to handling neighboring 3D image partitions. Each processor stores up to half the image data from each of its nearest neighbors in order to minimize communication overhead.

This domain decomposition allows efficient implementation of moving window operators such as median filtering. Furthermore, by performing such operations on the 3D image data, as opposed to multiple 2D image slices, we are able to use the full available 3D information to combat noise and ambiguity.

Operations such as cell boundary extraction can also be carried out efficiently. This is because the bulk of the inter-processor communications is between nearest-neighbor nodes on the 3D torus, for which dedicated hardware connectivity exists.

We assume that the image sequences are synchronously captured at a constant number of frames per second. Each processor can update its 3D volume data with the specific 3D image segment it is responsible for processing over the next time slice. The proposed domain decomposition will allow the imaging operations to be carried out effectively over large time-series data sets.

The domain decomposition is illustrated for the 2D case in Figure 7. Here, a 2D image is decomposed into 8 × 8 tiles. Consider the tile indicated by **R**. Suppose we are performing an operation such as recursive median filtering. At every iteration, the tile R may need to exchange data with its 8 nearest neighbors as indicated. This is because the computation of values within R may depend on the values of pixels within neighboring tiles. Similarly, Figure 8 illustrates the 26 nearest neighbors in three dimensions.

### MPI implementation

There are two phases involved at every step of an iterative algorithm, the first being communication and the second is computation.

To facilitate communication, we set up a 3D Cartesian communicator grid based on the number of processors available. Let us assume that each processor executes a single process. Data partitioning is then based on the process rank assigned by MPI. For communication, each node sends information to its nearest neighbors. This is implemented by using the *MPI sendrecv *command between appropriate pairs of processes. For instance, a process can send information to its neighbor to the north and receive information from its neighbor to the south. This procedure avoids deadlock during the computation. The data resident at each process is initialized by reading the appropriate partition from the disk.

Once each process has the required data, computation can proceed. Appropriate algorithms for image feature extraction and processing are implemented. This procedure of communication and computation is performed until all the data have been processed.

### Image processing algorithms

In order to process Denk and Horstmann's dataset, we used techniques published in the literature. The first processing step is to apply 3D median filtering [11]. This removes noise in the images while preserving the edges. We used a simple implementation for the 3D median filter, rather than more complex implementations as described in [12].

The second processing step is to extract contours corresponding to the neural structures, such as dendrites. There are many possible solutions to this problem, and the one we chose to implement is based on the concept of deformable templates, or snakes. We implemented the technique described by Xu and Prince [6]. Briefly, the snake is an energy-minimizing deformable shape, where the energy is a function of its internal elastic energy and an external force field. The force field is computed from the image data, e.g. the gradient of the image, as shown in Figure 3.

## Competing interests

ARR and GAC are salaried employees of IBM, which manufactures the Blue Gene/L supercomputer. They do not have any financial stake in sales of this machine. MM has no competing interests.

## Authors' contributions

ARR worked on problem formulation, initial design, followed by implementation and performance analysis. GAC worked on the problem formulation, solution design, and implementation. MM worked on problem identification, and design of the solution. All authors read and approved the final manuscript.
